# Impact of 1940s exposure to redlining on mortality and self-rated health later in life among older adults

**DOI:** 10.1101/2025.06.28.25330483

**Published:** 2025-06-30

**Authors:** Shuo Jim Huang, Dahai Yue, Kellee White Whilby, Michel Boudreaux, Rozalina G. McCoy, Kaitlynn S. Robinson-Ector, Neil J. Sehgal

**Affiliations:** 1. University of Maryland Institute for Health Computing, North Bethesda, MD, USA; 2. Department of Health Policy and Management, University of Maryland School of Public Health, College Park, MD USA; 3. Division of Endocrinology, Diabetes, & Nutrition, Department of Medicine, University of Maryland School of Medicine, Baltimore, MD, USA; 4. Division of Gerontology, Department of Epidemiology and Public Health, University of Maryland School of Medicine, Baltimore, MD; 5. Department of Health Systems and Population Health, University of Washington School of Public Health, Seattle, WA, USA

## Abstract

**Background:**

Historical redlining policies enacted in the 1930s and 1940s that restricted investment in Black neighborhoods shaped neighborhood conditions that may contribute to inequities in health and mortality among older adults today. Areas “redlined” by the Home Owners’ Loan Corporation (HOLC) in the 1930s against Black neighborhoods are associated with worse present-day area-level health outcomes. We examined whether early, personal exposure to redlining close to when the maps were drawn is associated with individual-level mortality hazard (survival time ratio) and self-rated health in older adults.

**Methods:**

We used mapped 1940 census enumeration districts to assign 1930s HOLC redlining categories (green A (“best”), blue B (“still desirable”), yellow C (“definitely declining”), and red D (“hazardous”)) to Health and Retirement Study participants based on 1940 census residence. We applied survey weights and ran a survival analysis with a parametric normal distribution maximum likelihood estimation to account for survivorship bias, and logistic regression on self-rated health, and included analyses stratified by race.

**Results:**

1940 HOLC-categorized yellow C (0.62 times the survival time, 95% CI: 0.41, 0.92) and red D (red: 0.59, 95% CI: 0.40, 0.87) exposures were significantly associated with reduced survival time compared to green A in both unadjusted and adjusted models. In stratified analyses, both Black and white residents of redlined areas had worse survival time ratios compared to green A, though the magnitude of effect was larger for Black residents than for white residents. Yellow C (Odds Ratio: 1.94, 95% CI: 1.16, 3.23) and red D (2.34, 95% CI: 1.37, 3.98) areas were also associated with increased odds of worse self-rated health compared to green A areas.

**Discussion:**

Living in redlined areas in the 1940s is associated with worse mortality survival for both Black and white older adults and with decreased self-rated health in older adults between 1992 and 2018. These findings extend beyond broader prior research demonstrating present-day area-level associations of redlining with worse health and are consistent with prior research on individual-level exposure to redlining. Associations with worse mortality in both Black and white residents (with stronger effects in Black residents) are consistent with theory and research demonstrating that structural racism degrades health for all communities.

## Introduction

Early life residential experiences—including exposures to substandard housing and impoverished neighborhoods^1–4^—have been shown to have strong associations with later life health outcomes and disparities across the life course^3–5^. These associations are driven by multiple overlapping place-based socio-economic and environmental factors^2^. Place-based factors persist over generations^6^ and are theorized to be caused by policies that entrench and retrench social and political hierarchies^2,6,7^ such as structural racism. One dimension of structural racism included historical redlining, a set of policies that systematically reduced economic access to predominantly Black neighborhoods, and named after color-coded maps drawn by the Home Owners’ Loan Corporation (HOLC)^8–11^.

In the late 1930s, the HOLC drew maps to categorize neighborhoods by perceived mortgage security risk using four color-coded categories: green A “best”, blue B “still desirable”, yellow C “definitely declining”, and red D “hazardous”^10,11^. The stark language used by the maps’ authors in notes on each neighborhood’s categorization explicitly referred to an area’s racial and ethnic composition in deeming an area “hazardous”^10,11^. While the HOLC maps were likely drawn too late to be used directly for its mortgage lending decisions^12^, they reflected prevailing racial attitudes towards specific neighborhoods that were then shared with the Federal Housing Administration (FHA)^10,12^. The FHA pursued housing policies that broadly excluded those predominantly Black neighborhoods from economic life^10^, leading to reduced investments in macroeconomic development and neighborhood environment conditions that lagged those in neighborhoods categorized by the HOLC as green A and blue B^10,11,13^. As a result, these HOLC “redlined” areas in the 1930s are still associated with worse socioeconomic outcomes in the present day^5,13–15^. The HOLC redlining maps therefore serve as a proxy for (and possible cause of^5,13,14^) a matrix of ongoing residential segregation policies throughout the 20^th^ century^10,12,13^.

Prior research has examined associations between 1930s HOLC redlining and present-day health outcomes^6^ at the area level (such as census tract) including cancer incidence and mortality, asthma severity, preterm birth, COVID-19 incidence, firearm mortality, and overall mortality including age-specific mortality and increased mortality in older age groups^3,15–26^. However, these previous area-level estimates of health effects associated with redlining may suffer from construct validity issues^27^ when area-level health constructs are applied to the individual-level and thus may misestimate the impact of redlining on individual-level health outcomes^24^. Additionally—given the use of both present-day residential exposure and present-day health outcomes—these studies can examine only the contemporaneous association of living in a redlined place today on population health at the same point in time, rather than how early exposure to redlining is associated with individual health later in life. A few studies have tracked individual rather than area-level outcomes^8,9,28^, but almost all of those^8,9^ also operationalized redlining exposure using present-day residents rather than historical residential exposure. Of the studies that tracked individual outcomes, only Linde and Egede have examined personal historical exposure to redlining to find increased mortality between 1988 and 2005 among those who lived in redlined areas in the 1940s^28^. That study, however, used only a subset of US cities^28^ where 1940 Census enumeration districts could be mapped with a particular, highly intensive method from the Urban Transition Project^29^, and as a result covered only 44% of the US 1940 urban population^30^. Thus, those findings are limited to only that subset of cities. Additionally, the study is limited by their use of only mortality without addressing quality of life, by lack of race-stratified analysis^6^, by lack of adjustment for average mortality selection given the gap between exposure and the start of data collection^31,32^, and by their use of continuous HOLC grade measures despite the non-interval nature of HOLC grades^33,34^. Connecting mortality (while accounting for average mortality selection and right censoring) as well as self-rated health outcomes to redlining status close to the 1930s in a larger set of cities using ordinal HOLC grades could allow us to better examine the role of initial redlining conditions on the health of older adults in the near present.

In this study, we aim to examine whether individual-level exposure to the early life conditions of redlining is associated with individual-level long-tail mortality and self-rated health outcomes. Using 1940 enumeration district, we merged historical 1930s redlining maps into a subset of respondents in the Health and Retirement Study (HRS) that had been linked to the 1940 Census^35^ (the first census conducted after redlining).

## Methods

We combined data from multiple historical, geographic, and longitudinal sources to code Health and Retirement Study (HRS) participants from 1992–2018 who were alive in 1940 with the HOLC redlining category of their residence in the 1940 census. We then used age at death and self-rated health at first HRS interview in the RAND longitudinal version of the HRS^36^ to analyze the association of 1940 redlining residence exposure with health outcomes experienced in later life.

The HRS is described in detail elsewhere^37^. Briefly, HRS recruits US adults over the age of 50 in birth period cohorts along with their spouses and oversamples Black participants and participants living in Florida. The study follows the same participants in waves every two years until they die or are otherwise lost to follow-up. We used the RAND longitudinal version of the dataset^36^ that matches participants consistently across waves from 1992 (wave 1) to 2018 (wave 14) and includes most core variables for each wave including self-rated health and age at death. We merged records from the restricted HRS 1940 Census complete count geographic dataset^35^ to the HRS RAND longitudinal file. Due to low variation in HOLC exposure categorization, we excluded race categorizations other than Black or white.

### Ethics statement

We sought ethics review from the University of Maryland Baltimore Institutional Review Board, which determined the work to be Not Human Subjects Research, and does not require informed consent. We also sought ethics review from the University of Maryland College Park Institutional Review Board, which determined the work to be Exempt, and does not require informed consent.

### Independent variable: HOLC category

We used the restricted HRS 1940 Census complete count geographic dataset^35^ that provided 1940 enumeration district information by matching HRS participants with the 1940 full count census housed by IPUMS USA^35^. This restricted 1940 full count geographic enumeration district level dataset has n=9602 HRS participants who were probabilistically matched to entries in the 1940 full count census^35^. Matched individuals were identified using a two-step process that initiated the matching process using a machine learning algorithm followed by multiple rounds of manually linking individuals^35^. There was a 95.1% agreement between matches identified manually and matches identified by the algorithm^35^. Matched individuals and households were coded with their 1940 enumeration district identifier^35^.

Enumeration districts allowed us to geographically link this subset of HRS participants to HOLC categories of each participant’s residence in 1940 using 1940 “virtual” enumeration districts^30^. We coded these “virtual” enumeration districts with HOLC category^30^ by predominance—largest overlapping HOLC category—after intersecting with the digital HOLC maps as georeferenced by the Mapping Inequality Project^11^. Our “virtual” enumeration districts were built algorithmically using address clustering^30^ from the Urban Transition Project’s Geographic Reference File^38^ alongside the Urban Transition Project’s city enumeration district shapefiles^29^. Our method included cities accounting for 84% of the USA’s 1940 urban population versus 44% for the Urban Transition Project’s city enumeration district shapefiles^30^ that were used by Linde and Egede^28^.

### Dependent variables

We operationalized mortality as survival time (reported using the survival time ratio compared to living in 1940 in a HOLC green A categorized neighborhood), using age in months at death. If a participant was alive at the end of wave 14 in 2018, we used their age in months at the end of the interview as a right censor indicator. Since some census-linked HRS participants were still alive at the end of the study period and right-censored, and to operationalize quality of life, we also used self-rated health as a secondary outcome. We used self-rated health at the first interview for a consistent measure for all study participants^39^. In accordance with common practice and the literature on how participants answer self-rated health^40^, we dichotomized the measure by grouping Excellent with Very Good; and Good, Poor, and Fair together.

### Covariates

We controlled for race (Black, white) in the unstratified adjusted models, as well as gender (male, female), birth year cohort, and birth region (census division) in all adjusted models. We did not control for marital status or insurance status due to their potential role as mediators^41^.

### Analysis Methods

We conceptualized the relationship of HOLC category exposure in 1940 with age at death in older age as a time-to-event/survival prospective study with left truncation. In this case, the left truncation occurred because of the lag time between the 1940 census and the start of the HRS. Since HRS is ongoing and a proportion of our sample is still alive, our study is also subject to right-censoring.

We used a parametric normal distribution maximum likelihood estimation regression model to account for both left truncation and right censoring^31^. Our variable entry time in the HRS (due to staggering both by HRS cohort and by age within each cohort) might suggest a non-parametric product limit estimator^31^. However, the near certain likelihood that left-truncated “observations” died earlier than the HRS inclusion criteria of age 50 would give us a biased estimator^31^ because of mortality selection and survivorship bias^32,42^ particularly given the large racial disparities in life expectancy throughout the 20th century^43^. Since racial discrimination also underpins redlining, it is plausible that many individuals from redlined areas may have died before they met the age-limited inclusion criteria for HRS, and thus those from redlined areas who survived long enough to enroll in HRS may have been especially healthy. If we looked only at the average life expectancy of those individuals we observed in HRS, those living in redlined areas may look as if they lived longer on average because our sample systematically missed all of those who had much shorter lifespans^42,44^. As a result, we may underestimate the impact of redlining. Instead, we used a parametric model since “adult” or non-neonatal age of death is close to normally distributed^45^. Accounting for left truncation in our survival analysis by modeling the shape of the distribution of “adult” death—which would include those who died before eligibility for HRS inclusion—should prevent attenuation of disparities like those found in other studies with older adults^44^ even with differential selection for truncation.

For the survival analysis, we estimated survival time ratio of survival time in each other HOLC category compared against survival time in green A areas. We estimated this survival time ratio first in an unadjusted model, followed by a fully adjusted model. For self-rated health, we performed a logistic regression of HOLC category on two levels of self-rated health and ran a second model with a full set of controls. In order to account for the HRS complex survey design, we applied person-level survey weights from each participant’s baseline year for all analyses. For both unadjusted and adjusted models of survival time ratio and self-rated health, we also stratified by race given the theoretical differences in Black-white racialized exposures to the effects of redlining^6^. We tested statistical significance at the 95% confidence level and report 95% confidence intervals. All statistical analyses were performed using Stata.

The study was determined exempt by the University of Maryland College Park Institutional Review Board and the University of Maryland Baltimore Institutional Review Board.

## Results

Our analytic sample consisted of 2926 HRS participants weighted to represent 7,834,211 population who were matched in the restricted 1940 Census geographic HRS dataset and who lived in a 1940 enumeration district that overlapped with HOLC categorized areas [Table 1]. 3.1% of our sample lived in green A “Best” enumeration districts, 15.1% in blue B “Still Desirable”, 43.5% in yellow C “Definitely Declining”, and 38.3% in red D “Hazardous” neighborhoods.

For those participants who died, mean age at death was 84 years. 46.4% rated their own health at the first interview as Excellent or Very Good, and 53.6% as Good, Fair, or Poor. The ratio between those who rated their health as Excellent or Very Good to those who rated their health as Good, Fair, or Poor reduced step-wise from green A enumeration districts to redlined D enumeration districts.

### Survival analysis

Our base unadjusted survival model found that living in 1940 in a redlined D enumeration district was statistically significantly associated with 0.59 times the survival time (95% CI: 0.38, 0.90) of living in green A areas [Table 2]. Yellow C enumeration districts were also significantly associated with worsened survival time (0.64, 95% CI: 0.41, 1.00). When controls are added, red D (0.59, 95% CI: 0.40, 0.87) and yellow C (0.62, 95% CI: 0.41, 0.92) categories continued to be significantly associated with worsened survival time ratios compared to green A.

In analysis stratified by race, Black participants living in red D areas had 0.17 times the survival time (95% CI: 0.13, 0.22) compared to those living in HOLC green A areas. White participants living in redlined areas had 0.62 the survival time (95% CI: 0.40, 0.95) of those living in green A. The direction and significance of these results were unchanged with the addition of controls. Additionally, Black participants living in yellow C neighborhoods also had worsened survival times in both unadjusted (0.14, 95% CI: 0.06, 0.33) and adjusted models (0.13, 95% CI: 0.03, 0.72). After adjusting for controls, white participants living in yellow C neighborhoods also had worsened survival times (0.62, 95% CI: 0.42, 0.92). Black participants living in blue B neighborhoods had worsened survival times (0.23, 95% CI: 0.09, 0.58) in the unadjusted model, but not the adjusted model.

### Self-rated health

For self-rated health, yellow C and red D enumeration districts were significantly associated with decreased self-rated health [Table 3]. Red D was associated with 2.33 increased odds (95% CI: 1.39 to 3.91) of reporting only Good, Fair, or Poor health. Yellow C was associated with 1.92 increased odds (95% CI: 1.16 to 3.17) of reporting only Good, Fair, or Poor health.

When controls are added, red D enumeration districts continued to be associated with worsened self-rated health, with 2.34 increased odds (95% CI: 1.37, 3.98) of reporting only Good, Fair or Poor health. Yellow C enumeration districts also continued to be associated with worsened self-rated health (1.94, 95% CI: 1.16, 3.23).

There was quasi-complete separation for Black participants for logistic regression for red D and green A areas. For white participants, red D (2.13, 95% CI: 1.25, 3.62) and yellow C areas (1.90, 95% CI: 1.14, 3.15) were both associated with higher odds of worse self-rated health compared to green A areas in both unadjusted and adjusted models.

## Discussion

To understand the association of individual experience residing in redlined areas in the 1940s with mortality and quality of life in older age, we analyzed survival time ratios and odds of worse self-rated health by HOLC grade of HRS participants’ residence in 1940 enumeration districts. We examined the association with individuals who were exposed shortly after redlined maps were drawn and found that redlined D “hazardous” enumeration districts were associated with both poorer survival time and worse self-rated health in older age in both our unadjusted and fully adjusted models. These results demonstrate that early life experiences with structural racism through living in redlined areas are associated with worse health outcomes across the life course. Additionally, our findings help to inform whether redlining’s associations with area health outcomes are due solely to the concentration of impoverished populations^46^ or are partly due to exposures over the life course^47,48^.

In race-stratified analyses, both Black and white participants living in 1940 in red D neighborhoods had worse survival times compared to those living in green A neighborhoods. However, the ratio of difference in survival time for Black participants in redlined D neighborhoods compared to HOLC green A neighborhoods was worse than that for white participants by a factor of three. While we were not able to conduct stratified analyses for Black participants for self-rated health due to quasi-complete separation, we similarly found worsened self-rated health for white participants in red D versus green A neighborhoods. This finding lends support to current literature and theory that structural racism harmed and continues to harm the health of both white and Black residents^49,50^, even while the worst effects are concentrated in Black residents^49^.

Previous scholarship has found that redlined areas were associated at both area^3,6,15–26^ and individual levels with worse health outcomes for present-day residents^8,9^, including on mortality and life expectancy. We found that these associations exist not just for present-day residents but also those who were residents when these areas were first redlined after the late 1930s, regardless of where they lived at follow-up. Our findings are also consistent with Linde and Egede’s recent finding in a smaller subset of large US cities (covering 44% of the US 1940 urban population) that living in 1940 in a redlined area was associated with early mortality later in life^28^. We increased the set of cities to include cities covering 84% of the US 1940 urban population and find a similar pattern with reduced quality of life as well. We also treated HOLC grades as ordinal (and non-interval) rather than continuous to better match the non-monotonic sociological distance between HOLC grades.

Finally, we found an association between yellow-lining and mortality and self-rated health. Yellow-lining was first explored for economic outcomes by Aaronson et al., where areas that were categorized yellow C “Definitely Declining” by the HOLC also led to poor socioeconomic outcomes in addition to those found in redlined D areas^14^. This important finding reinforces our previously demonstrated association between yellow-lining and area-level present-day health outcomes in Baltimore^24^.

Early life has been identified as a key period in setting trajectories of health^3,4,6,8^. Initial early life exposures in housing and neighborhoods may have stronger associations with health for older adults compared to exposures later in adulthood due to how they act on childhood development, whether through physical environment—such as lead exposure^51^—or social environment—such as Adverse Childhood Experiences^52^. Our findings are broadly consistent with such findings that show that earlier in life exposure to the racialized construction of geographic space^5,16^ is associated with poorer health outcomes later in the life course.

### Future Directions

Future analysis should examine outmigration—whether and what proportion of individuals still live in the same HOLC grade as they did in 1940—to explore whether associations found on the individual level are more associated with initial or continued exposure to areas redlined in the 1930s, and whether present-day area level effects in redlined neighborhoods are more associated with population change or lack of resources and investment. Other scholars have theorized that over the long-term, residents in racially segregated and resource-deprived neighborhoods rely on community trust, social solidarity, and mutual resources to somewhat mitigate infrastructure deprivation, and thus the displacement of long-term residents from redlined areas due to economic redevelopment schemes then may worsen these former residents’ health^53,54^. Alternatively, moving to low-poverty neighborhoods has been associated with age-dependent educational and economic gains^55^ and reduced hospitalizations^56^. Leveraging data on resident mobility would allow researchers to test these theories in the context of redlining.

Future analyses might also utilize individual address level, since we know georeferenced HOLC categories with far greater certainty than we do enumeration districts, and since HOLC categories are non-cartographic with respect to any other cartographic division. Exact address matching would also enable the inclusion of other measures of interest that are strongly associated with health outcomes from a young age. However, due to changes in city geographies^29^, exact address matches should be used with caution or snapped to an enumeration district before a dataset of georeferenced 1940 addresses is constructed (rather than simulated from present-day addresses).

Many of the antecedent conditions to redlining stretch to the early postbellum or possibly even antebellum period^57^. Areas where new city arrivals lived (many of whom were Black and/or immigrants) often contained the oldest housing stock^12,38,57^. Additional work could be done to link histories of place^25,58^, forced migration, and compulsory labor throughout the 19th and 20th centuries to help us understand the health landscape in the present day. Our method of measuring exposure to redlining could also be used to connect a larger proportion of the 1940 full count census to vital records or other individual level datasets.

### Policy Implications

The history of redlining as policy may create reparative responsibilities for governance. Institutional, local, state, and national governmental policies created a set of ongoing conditions (likely deliberately) through political disenfranchisement that continue to rob segments of the population of the full length and quality of life that they are owed^10–12,14,51^. Additional investment in both funding specified for formerly redlined areas and residents and for broad-based social investments are also warranted to reverse decades of population-level harms from structural racism^1^. We found that redlining is associated with worse longevity and health for both Black and white residents. As the US government in 2025 calls for health research that only benefits “all” people rather than specific populations, it is essential to recognize that addressing health equity and the specific histories of racial exclusion in fact does benefit all people.

### Limitations

We could not observe how long HRS participants lived in redlined areas. Migration and different exposure time to redlining may have caused bias in our estimates. The future release, cleaning, and linking of additional full-count decadal censuses past 1940 to HRS may become an opportunity to control for length of exposure to redlining.

Enumeration districts are area constructions that do not align perfectly with the non-cartographic categorization of HOLC areas^30^. However, enumeration districts do have a much higher resolution due to their smaller size and geographic compactness than larger geographic units such as census tracts or city-defined neighborhoods, particularly in dense urban areas^30,59^. Some participants missing HOLC-categorized enumeration districts may have lived in enumeration districts excluded by the precursor Geographic Reference File dataset by population size (smaller incorporated cities)^38^. However, the HOLC did not draw maps for most smaller cities^11,14^.

HOLC categorizations were not necessarily a unified sociological construction among different cities/metropolitan areas^6,11^. As a power hierarchy, the racial hierarchy in the US is not an internally consistent construct but rather may vary in its porousness or the saliency of certain “rules” within each local context to maintain that power structure^60^. As such, the decision to mark certain similar areas as red, yellow, blue, or green reflected local racial attitudes and thus varied within the general bounds of American racial policy from city to city^11,12^. Additionally, local political contexts are likely to have determined how resources and investments were allocated to and from politically marginalized communities from 1940 to the present day^11^. For this study, we treated HOLC categories as if they were consistent across cities.

Although some studies have found a causal effect from the categorization used in the HOLC maps^14^ on economic and social outcomes in the 20^th^ and 21^st^ centuries, the maps themselves are more broadly treated as having largely labeled and codified the existing real estate practices and urbanization trajectories that had resulted in racialized patterns of residential settlement from before the 1930s^12,57^, and thus as proxies for a matrix of redlining policies. Our study design is unable to differentiate among any effects of the maps themselves, underlying residential patterns, or redlining policies throughout the 20^th^ century.

### Conclusion

Direct personal experience of living in a HOLC redlined area in the 1940s is associated with worse mortality and worse self-rated health later in life between 1992 and 2018.

## Figures and Tables

**Table 1:** Summary statistics at first HRS interview

Variable	Total	HOLC A (Green: Best)	HOLC B (Blue: Still Desirable)	HOLC C (Yellow: Definitely Declining)	HOLC D (Red: Hazardous)	p-value
**Total HRS RAND sample**	42233					
**1940 Census Geo HRS sample**	9602					
**Analytic sample**	2926					
**HOLC categories**		3.1%	15.1%	43.5%	38.3%	
**Year of birth**	1925 (0.3)	1924 (1.4)	1924 (0.5)	1925 (0.4)	1926 (0.5)	0.0321
**Age at first interview**	69 (0.3)	70 (1.4)	70 (0.5)	69 (0.4)	68 (0.5)	0.0374
**Age at death**	84 (0.2)	87 (1.2)	85 (0.5)	85 (0.4)	83 (0.4)	0.0042
**Died during study period**						
Right Censored	30.3%	35.6%	31.1%	29.6%	30.5%	
Died	69.7%	64.4%	68.9%	70.4%	69.6%	0.7421
**Self rated Health 1st Interview**						
Excellent or Very Good	46.4%	63.1%	51.4%	47.1%	42.4%	
Good, Fair, or Poor	53.6%	36.9%	48.6%	52.9%	57.6%	0.0018
**Race**						
white	94.6%	99.4%	97.6%	99.0%	88.0%	
Black/African American	5.4%	0.6%	2.4%	1.0%	12.0%	<0.0001
**Gender**						
male	43.9%	47.4%	44.8%	44.7%	42.4%	
female	56.1%	52.6%	55.2%	55.3%	57.6%	0.6247

HRS: Health and Retirement Study. HOLC: Home Owners’ Loan Corporation, referring to residence in that category in the 1940 census.

**Table 2:** Survival analysis: survival time ratio by HOLC grade

	All			Stratified Black			Stratified White		
	Time Ratio	95% CI		Time Ratio	95% CI		Time Ratio	95% CI	
**Unadjusted model**									
**HOLC**									
A (green)	ref			ref			ref		
B (blue)	0.73	0.47	1.15	*0.23	0.09	0.58	0.75	0.48	1.16
C (yellow)	*0.64	0.41	1.00	*0.14	0.06	0.33	0.65	0.41	1.01
D (red)	*0.59	0.38	0.90	*0.17	0.13	0.22	*0.62	0.40	0.95
**Adjusted model**									
**HOLC**									
A (green)	ref			ref			ref		
B (blue)	0.72	0.48	1.08	0.20	0.03	1.30	0.72	0.49	1.08
C (yellow)	*0.62	0.41	0.92	*0.13	0.03	0.72	*0.62	0.42	0.92
D (red)	*0.59	0.40	0.87	*0.17	0.03	0.89	*0.59	0.40	0.87
**Race**									
white	ref			N/A			N/A		
Black	*0.67	0.52	0.86						
**Gender**									
male	ref			ref			ref		
female	*1.70	1.52	1.91	*1.89	1.12	3.21	*1.69	1.51	1.89

Time ratio: reported as time survived as a ratio of reference group per unit increase. HOLC: Home Owners’ Loan Corporation.

*: statistically significant at 95% confidence level. Adjusted models also controlled for birth year and census region of birth (both suppressed to preserve anonymity).

**Table 3:** Worse self-rated health (dichotomized) at Health and Retirement Study entry

	All				Stratified White			
	Odds Ratio		95% CI		Odds Ratio		95% CI	
**Unadjusted model**								
**HOLC**								
A (green)	ref				ref			
B (blue)		1.62	0.99	2.64		1.56	0.94	2.56
C (yellow)		*1.92	1.16	3.17		*1.90	1.14	3.15
D (red)		*2.33	1.39	3.91		*2.13	1.25	3.62
**Adjusted model**								
**HOLC**								
A (green)	ref				ref			
B (blue)		1.56	0.93	2.60		1.53	0.91	2.58
C (yellow)		*1.94	1.16	3.23		*1.94	1.15	3.27
D (red)		*2.34	1.37	3.98		*2.36	1.37	4.05
**Race**								
white	ref				N/A			
Black		*1.96	1.35	2.84				
**Gender**								
male	ref				ref			
female		0.92	0.78	1.08		0.91	0.77	1.07

HOLC: Home Owners’ Loan Corporation.

*: statistically significant at 95% confidence level. Adjusted models also control for birth year and census region of birth (both suppressed to preserve anonymity).
